# Mechanism of allergic rhinitis treated by *Centipeda minima* from different geographic areas

**DOI:** 10.1080/13880209.2021.1923757

**Published:** 2021-05-19

**Authors:** Yanzhuo Jia, Junbo Zou, Yao Wang, Xiaofei Zhang, Yajun Shi, Yulin Liang, Dongyan Guo, Ming Yang

**Affiliations:** a Department of Pharmaceutics, Shaanxi University of Chinese Medicine, Xianyang, China; b Department of Pharmaceutics, The Key Laboratory of Basic and New Drug Research of Traditional Chinese Medcine, Shaanxi University of Chinese Medicine, Xianyang, China; c Department of Pharmaceutics, Jiangxi University of Traditional Chinese Medicine, Nanchang, China

**Keywords:** Network pharmacology, volatile oil, molecular docking

## Abstract

**Context:**

The coriander plant *Centipeda minima* (L.) A. Braun et Aschers (Compositae) is used for the treatment of allergic rhinitis.

**Objective:**

Analyze the difference of the *C. minima* volatile oil from 7 geographic areas and its therapeutic effect on allergic rhinitis.

**Materials and methods:**

The volatile oils from different geographic areas were extracted and analyzed, the protein and biological pathway for the treatment of allergic rhinitis were predicted by network pharmacology. Established three groups of Sprague-Dawley rat allergic rhinitis models (n = 10). The treatment group was given 100 μL/nostril of 0.1% *C. minima* volatile oil, the blank and model groups were given the same amount of normal saline. After 15 days, serum inflammatory factors were detected by ELISA. Nasal mucosa tissues were examined by hematoxylineosin staining and immunuhistrochemistry.

**Results:**

There are differences in the content of volatile oil in the seven geographic areas. Experiments showed that the concentration of TNF-α in the serum of the administration group decreased from 63.66 ± 2.06 to 51.01 ± 4.10 (pg/mL), IL-4 decreased from 41.90 ± 3.90 to 28.68 ± 3.39 (pg/mL), IgE decreased from 22.18 ± 1.40 to 17.59 ± 1.60 (pg/mL), IL-2 increased from 314.14 ± 10.32 to 355.90 ± 10.01(pg/mL). Immunohistochemistry showed that compared with the model group, the PTGS2 and MAPK3 proteins in the administration group were significantly reduced.

**Discussion and conclusions:**

*C. minima* volatile oil is a multi-target and multi-pathway in the treatment of allergic rhinitis, which provides a new research basis and reference for the treatment of allergic rhinitis.

## Introduction

The coriander plant *Centipeda minima* (L.) A. Braun et Aschers (Compositae), described on the (http://www.theplantlist.org) website, is also known as chickweed. The plant is drought-resistant and grown in most provinces of China. *C. minima* has a spicy taste and has been shown to minimize nasal secretions and cough associated with colds and other respiratory complications (Jia and Zhang 2018). It has been demonstrated that the main medicinal chemical components of *C. minima* include volatile oils, flavonoids, and polysaccharides (Cao and Li 2012). Notably, the volatile oil components in *C. minima* were suggested to be the main anti-inflammatory effectors of this medicinal plant (Zhang et al. 2013). Accordingly, those components were traditionally used for the treatment of allergic rhinitis and associated headache, although the underlying therapeutic mechanism of these medicinal effects remains unclear.

Allergic rhinitis is a non-infectious inflammatory disease that affects the nasal mucosa. It has been marked by paroxysmal sneezing, runny and itchy nose, and nasal congestion. These complications can impact work and other daily activities. Studies have shown that *C. minima* can significantly inhibit the activation of eosinophils and mast cells, reduce pathological changes in nasal mucosal tissues, reduce histamine levels, and lessen nasal obstruction (Liu et al. 2005; Gao & Mei 2010).

In this experimental study, volatile oil components were extracted from *C. minima* collected in seven different geographic areas of China. The best extraction conditions were experimentally determined using steam distillation. Following extraction, gas chromatography-mass spectrometry (GC-MS) was used to analyze the volatile oil composition of *C. minima*. Network pharmacology analysis was used to explore component-related molecular targets. The overall level of protein-disease correlation was assessed and the main pathways and key targets of *C. minima* components were determined. Molecular docking tests were done using the identified target proteins. The *C. minima* was used in *in vivo* animal experiments to evaluate drug efficacy and provide a reference for further clinical experiments.

## Materials and methods

### Extraction of volatile oil from *C. minima*

The whole herb of *C. minima* was collected in seven producing areas of China including Guangdong, Henan, Sichuan, Jiangxi, Guangxi, Hubei, and Shanxi. All collected specimens were purchased from Tiandi Network of Traditional Chinese Medicine (batch number 20200222). The plants were identified as *C. minima* by Professor Yonggang Yan of Shaanxi University of Traditional Chinese Medicine. First, the best oil extraction process was determined by steam distillation. The mesh size number was changed and the oil yield of medicinal materials powder was tested by sieving through 10, 20, 40, 60, 80, and 100 mesh. The oil yield was obtained when the amount was 6, 8, 10, 10, and 12 times the amount of water oil. The oil yield was obtained at 220 °C, 240 °C, 260 °C, 280 °C, and 300 °C, respectively, as described previously (Li et al. 2006). Following this, volatile oils and other components were collected from different *C. minima* plants obtained in seven geographic locations for further analysis.

### Determination of chemical composition of volatile oils from *C. minima*

The chemical composition of the extracted volatile oil from *C. minima* was estimated using GC-MS. The GC-MS included Agilent HP-5ms (30 m × 250 μm × 0.25 μm) capillary column. The used carrier gas was high purity He. The used injection volume was 3 μL. The split ratio was 50:1. The flow rate was set at 1 mL/min. Temperature program was set as follows: initial temperature was 55 °C with gradual temperature change set as 8 °C/min; reaching 80 °C hold for 1 min; the following gradual temperature change was set as 6 °C/min; reaching 200 °C hold for 3 min; the following gradual temperature change was set as 3 °C/min; reaching 250 °C hold for 3 min. Mass spectrometry conditions were set as following: EI was used as an ion source; electron energy was 70 eV; ion source temperature was 230 °C, MS quadrupole temperature was 150 °C; multiplier voltage was 1.5 kV; scan mass range was 28∼555 *m/z*; scanning interval was 0.5 s; and scan speed was 781 amu/s as described previously (Liu et al. 2002; Tang et al. 2019). The *n*-alkane standard (C7-C40) was purchased from Jiangxi Up-style Biotechnology Co., Ltd. The standard solution was analyzed under the described GC-MS conditions. The retention index of each component was calculated according to the standard data using the following formula

RI=100n+100[tR(x)−tR(n)]/[tR(n+1)−tR(n)]

where tR (x), tR (*n*), and tR (*n* + 1) represent the retention time of the *n*-alkane to be measured, the carbon number *n*, and the carbon number *n* + 1, respectively. It was considered that tR (*n*) <tR (x) <tR (*n* + 1) as described previously (Zhang et al. 2019).

### Fingerprint and cluster analysis

The GC-MS data were processed using Data Analysis software. For similarity analysis, the data was imported into the similarity evaluation system of chromatographic fingerprint for traditional Chinese medicine as described previously (Liu et al. 2018; Lin et al. 2019). To detect the differences between the ingredients from plants collected in different geographic areas, SIMCA software was used to perform a cluster analysis on the content of ingredients.

### Acquisition and comparison of *C. minima* component target with the targets of allergic rhinitis

Data Analysis, the common components of the seven geographical regions were obtained using Venny 2.1.0 (https://bioinfogp.cnb.csic.es/tools/venny/index.html). To identify targets related to the *C. minima* composition, we used Pubchem (https://pubchem.ncbi.nlm.nih.gov/) search for the CAS (Chemical Abstracts Service). Then the Canonical SMILES numbers were copied and pasted on the Swiss Target Prediction (http://www.swisstargetprediction.ch/) website tool. We used websites such as DisGeNET (http://www.disgenet.org/), OMIM (https://omim.org/), TTD (https://db.idrblab.org/ttd/) and Genecards (https://www.genecards.org/) to find allergic rhinitis targets (Xiaoxiao et al. 2019; Nong et al. 2020).

### *C. minima* component-related targets and associated allergic rhinitis target mapping

To obtain intersection targets of the volatile oil components and rhinitis, the identified *C. minima* component targets and allergic rhinitis-related targets were imported into Venny 2.1.0 software. The intersection target was regarded as the effective protein-target of *C. minima* component linked to allergic rhinitis. Using the Merge function of Cytoscape 3.7.1 software, the component-target-disease network map was constructed. On the map, nodes represent components, targets, and diseases; edges were used to connect components, targets, and diseases. The *C. minima* protein targets in allergic rhinitis were analyzed using the constructed network.

### Protein interaction network

As described above, the identified intersection proteins were uploaded to the STRING platform (https://string-db.org) (Tao et al. 2020). Within this software tool, the data were uploaded as Multiple proteins to obtain the protein interaction network figure. The output result was set in TSV format, and uploaded to Cytoscape 3.7.1 software. Cytoscape analysis was conducted to get the topology characteristics of the network and adjust the node size according to the Degree value.

### GO analysis and KEGG analysis

Using R language (Guangchuang et al. 2012), we analyzed the common genes associated with of *C. minima* and allergic rhinitis using GO (GeneOntology) and KEGG (Kyoto Encyclopedia of Genes and Genomes) tools. Pathway enrichment data was collected to reflect the biological effect of *C. minima* on allergic rhinitis.

### Molecular docking

The top three proteins were selected according to their protein degree value and betweenness centrality calculated using topological network analysis with Cytoscape software. The analyzed value represents the number of connections between the node and other nodes, and the median value is the ratio of the number of paths passing through the node to the total number of shortest paths in the network (Zhang et al. 2021). Those values are an important topological parameter in the network. Following this, we found positive drugs corresponding to the protein using Drugbank. The positive drugs and their corresponding *C. minima* components as ligands were downloaded using PDB format of protein 3D structure and SDF format of ligand 2D structure. Molecular docking was analyzed using Discovery Studio 4.0 software. Firstly, the 3D structure of the protein was imported into the software and water groups were deleted. Following this, conventional procedures were performed to complete the incomplete residues, delete the excess protein conformation, hydrogenate, and distribute the relevant charges. The complete ligand 2D structures were imported into the software. After basic preprocessing, the software was used to identify the protein active centers, select the (Libdock) docking mode, define default docking parameters, and analyze the data (Yao et al. 2019).

### Allergic rhinitis animal models *in vivo*

Sprague Dawley SPF grade male rats (180 ± 20 g) were purchased from Chengdu Dashuo Experimental Animal Co., Ltd. (production license certificate number SYXK (Chuan) 2015-030). The study protocol was approved by the animal ethics committee for Shaanxi University of Chinese Medicine. Before the experiment, the rats had free access to food and drink under the circadian rhythm light conditions for 7 days. Thirty rats were randomly divided into 3 groups. All groups, except the blank control group, were injected with ovalbumin OVA (purchased from Sigma) and aluminum-containing adjuvant to initiate allergic rhinitis model according to the previously described method (Liu et al. 2013; Salimi et al. 2019). After successful establishment of the model, the rats in the blank group and model group were given normal saline in nasal cavity every day. The rats in the treatment group were given 100 μL/nostril of 0.1% *C. minima* volatile oil. To observe the pathological changes after 15 days treatment, the animals were humanely euthanized, their nasal mucosa tissues were cut out, fixed with 4% paraformaldehyde for 24 h, and processed using conventional dehydration method, paraffin embedding, sectioning, and hematoxylin-eosin (H&E) staining.

### Determination of TNF, IL-2, IL-4, and IgE levels using enzyme-linked immunosorbent assay (ELISA)

To detect the inflammatory factors, ELISA kits for anti-rat TNF, IL-2, IL-4, and IgE (were purchased from Shanghai Yuchun Biotechnology Co., Ltd. (batch number: 20201116). The isolated rat serum was extracted and operated according to the manufacturer’s instructions. Absorbance values were determined at the 450 nm wavelength. ELISA standard was drawn to calculate the contents of TNF, IL-2, IL-4, and IgE in the serum according to the manufacturer’s instructions.

### Immunohistochemistry

After the pathological tissue was ice-sectioned to room temperature, fixed with 4% paraformaldehyde for 20 min, after antigen retrieval, incubated with 3% H_2_O_2_ at room temperature for 25 min, washed 3 times with PBS, 5 minutes each time, added the first antibody after serum blocking, overnight at 4 °C, washed with PBS After 3 times, add secondary antibody dropwise, incubate at room temperature for 50 min, rinse with PBS, stain with DAB, control staining time under the microscope, counterstain with hematoxylin, dehydration with gradient alcohol after washing, transparent xylene, mount with neutral resin, observe the result under microscope. Use Image-Pro Plus image analysis system to analyze protein expression and average optical density value (Watanabe et al. 2018).

### Statistical analysis

The experimental data were expressed as the mean ± standard deviation, and the one-way analysis of variance ANOVA is used to compare more than two groups.

## Results

### The best conditions for volatile oil extraction

**Figure 1.**
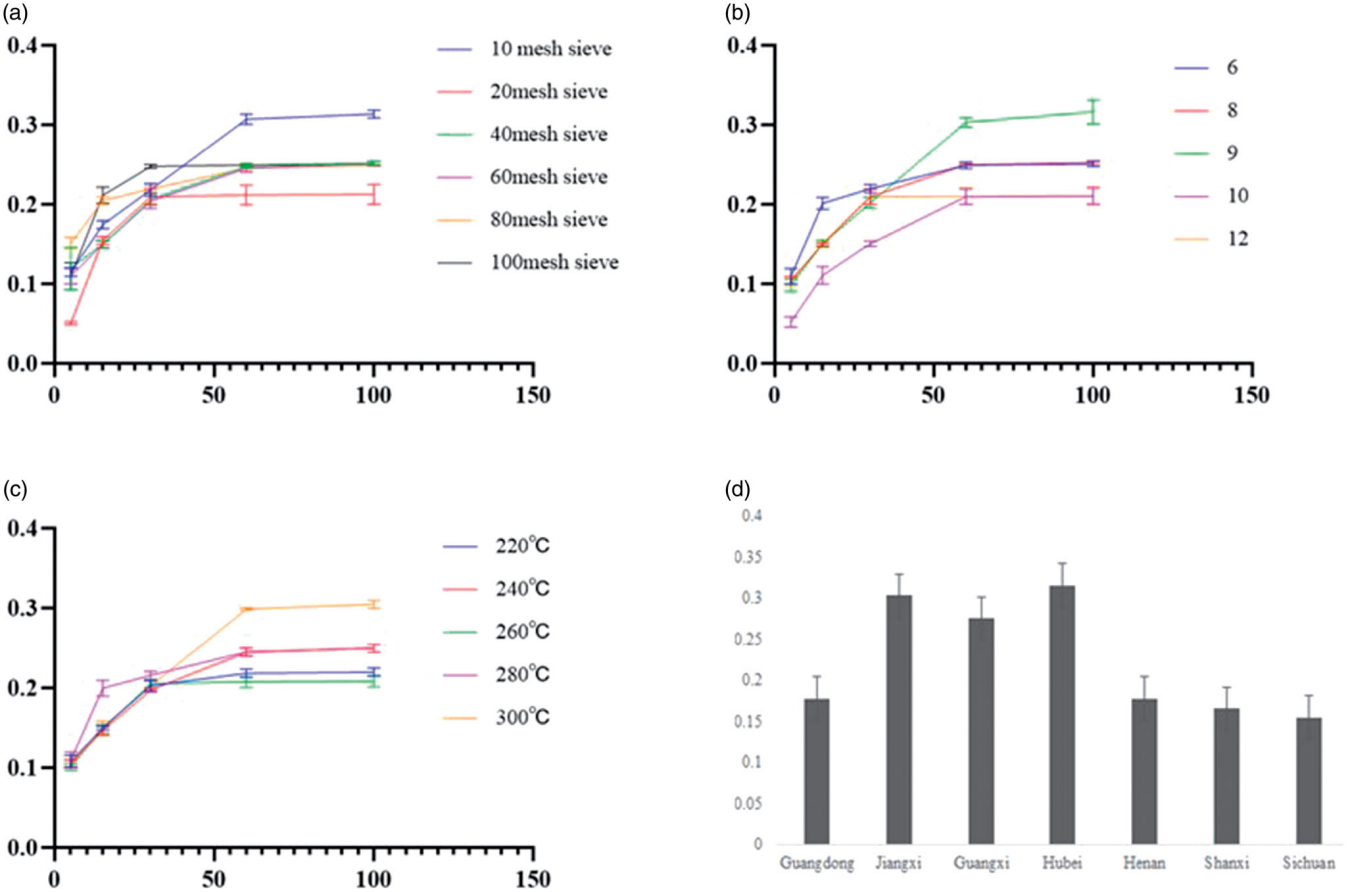
Extract results. (a) Number of mesh sieve. (b) Added water quantity. (c) Heating temperature. (d) Extraction rate from different geographic areas.

The best extraction conditions were chosen as shown in Figure 1(a–c), During the extraction, samples were passed through a 10-mesh sieve, 9 times the amount of water was added. *C. minima* oil yield was the highest at 300 °C The oil extraction rates from plant collected at different geographic areas are shown in Figure 1(d).

### Fingerprint and cluster analysis

**Figure 2.**
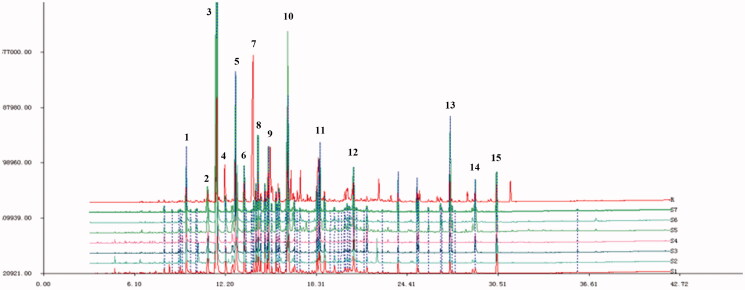
Fingerprints of volatile oil of *C. minima* from different producing areas (S1 Shanxi; S2 Hubei; S3 Jiangxi; S4 Guangxi; S5 Henan; S6 Sichuan; S7 Guangdong).

**Figure 3.**
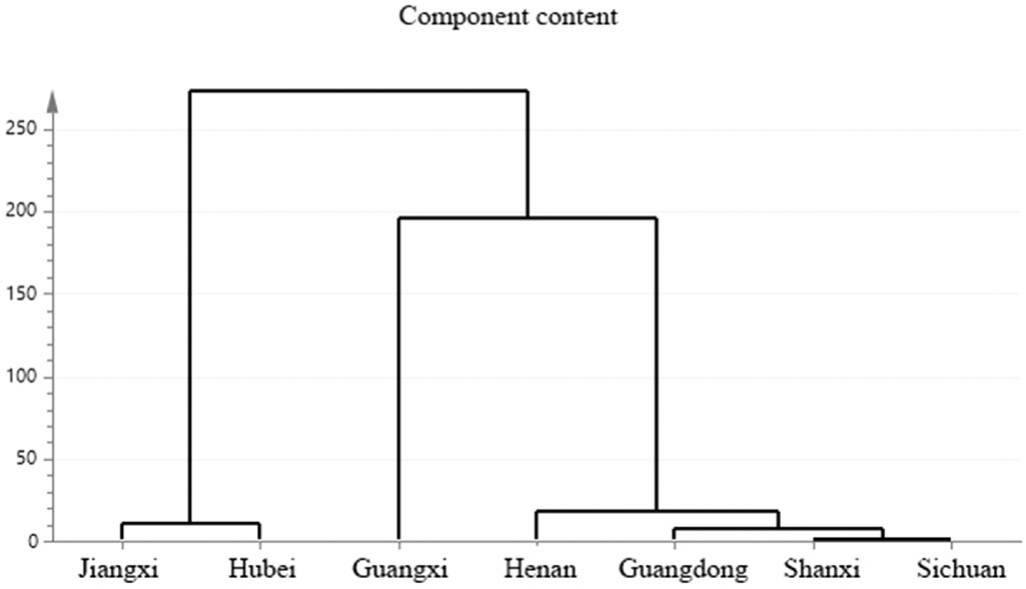
Cluster analysis diagram.

*C. minima*-extracted volatile oil-related GC-MS data were imported into the similarity evaluation system of chromatographic fingerprints of traditional Chinese medicine. The obtained fingerprints are shown in Figure 2. The similarity analysis data is shown in Table 1. The data shows small differences between *C. minima* composition samples collected from seven geographic areas. Notably, Shanxi and Hubei, Shanxi and Sichuan, Jiangxi and Guangxi, Shanxi and Guangdong samples were found as similar in composition (similarity from 0.90 to 0.98). Shanxi and Henan, Henan and Guangdong, Hubei, and Guangxi were found similar in composition (similarity from 0.82 to 0.90 range). The *C. minima* component cluster analysis data is shown in Figure 3. The results indicate that the content of plants collected in Jiangxi and Hubei, Shanxi and Sichuan, is relatively similar. According to the data analysis, we identified about 30 types of volatile oil *C. minima* compounds for each region, of which 15 compounds were identical among all plants. The highest levels of bicyclo[3.1.1]hept-2-en-6-ol, 2,7,7-trimethyl-, acetate, [1S-(1.α.,5.α.,6.β.)]- were detected in all samples, although Henan area plants indicated the lowest relative content and Guangxi has the highest content of the compound. The content of butanoic acid, 2-methyl-, 3,7-dimethyl-2,6-octadienyl ester, (*Z*)-compound was not much different in all regions, but the lowest content of this compound was observed in Sichuan area. The content of cis-chrysanthenol was the lowest in Henan and Guangdong areas compared to other regions.

**Table 1.** Fingerprints of volatile oil of *C. minima* from different producing areas.

	S1	S2	S3	S4	S5	S6	S7	Comparison fingerprint
S1	1.000	0.973	0.926	0.898	0.825	0.981	0.977	0.976
S2	0.973	1.000	0.910	0.888	0.805	0.964	0.958	0.964
S3	0.926	0.910	1.000	0.981	0.927	0.920	0.905	0.980
S4	0.898	0.888	0.981	1.000	0.859	0.898	0.861	0.952
S5	0.825	0.805	0.927	0.859	1.000	0.808	0.852	0.909
S6	0.981	0.964	0.920	0.898	0.808	1.000	0.952	0.969
S7	0.977	0.958	0.905	0.861	0.852	0.952	1.000	0.965
Comparison fingerprint	0.976	0.964	0.980	0.952	0.909	0.969	0.965	1.000

Table note: S1 Shanxi; S2 Hubei; S3 Jiangxi; S4 Guangxi; S5 Henan; S6 Sichuan; S7 Guangdong.

### *C. minima* and allergic rhinitis target prediction

**Figure 4.**
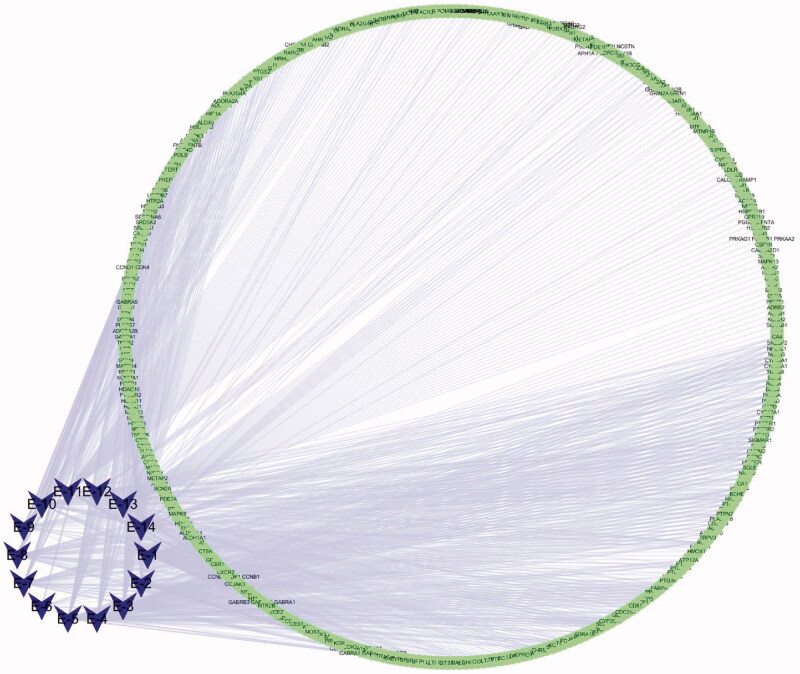
Component-target diagram.

We extracted 15 common volatile oil constituents from *C. minima* specimens collected in seven geographic areas using Venny software tool. The 15 constituents and their total percentages (Pct Total) detected for each geographic region is shown in Table 2, the peak number of Figure 2 also marks the common components in the order of the table. After database search, a total of 343 related targets for 15 components and 2155 disease targets were obtained. The component-target diagram is constructed by using Cytoscape software, as shown in Figure 4.

**Table 2.** Components and Pct Total in each production area.

NO.	compound	RT	Sichuan	Henan	Guangdong	Jiangxi	Hubei	Guangxi	Shanxi
1	cis-Chrysanthenol	1092.16	4.516	0.834	1.538	4.232	3.484	4.141	5.889
2	8,9-Dehydrothymol	1103.209	1.389	1.986	2.185	1.786	1.721	1.017	1.989
3	Bicyclo[3.1.1]hept-2-en-6-ol, 2,7,7-trimethyl-, acetate, [1*S*-(1.alpha.,5.alpha.,6.beta.)]-	1234.799	34.797	16.547	23.552	31.082	28.231	39.184	27.953
4	Modephene	1393.533	0.937	1.155	1.536	0.881	1.513	0.899	1.343
5	Petasitene	1401.937	0.475	0.719	0.944	0.663	0.653	0.657	0.805
6	.beta.-isocomene	1414.405	1.051	1.235	1.728	0.961	1.083	0.94	1.525
7	Caryophyllene	1417.262	3.31	3.101	3.763	3.482	3.214	3.299	3.28
8	*trans*-.alpha.-Bergamotene	1438.626	0.328	0.341	0.486	0.266	0.328	0.22	0.403
9	alpha.-Humulene	1452.529	0.561	0.994	1.889	0.751	0.851	0.669	0.863
10	10*S*,11*S*-Himachala-3(12),4-diene	1426.106	2.341	3.723	5.028	4.36	3.707	3.097	3.665
11	Copaene	1574.242	1.191	0.417	0.722	0.457	0.714	0.339	0.732
12	Butanoic acid, 2-methyl-, 3,7-dimethyl-2,6-octadienyl ester, (*Z*)-	1583.259	0.516	6.33	5.351	1.405	2.647	1.282	3.153
13	Cyclododecane	1678.479	3.096	2.289	3.51	3.043	3.592	3.324	3.388
14	2-Pentadecanone, 6,10,14-trimethyl-	1866.45	2.482	1.208	0.718	0.348	0.141	0.434	0.702
15	Docosane	2494.165	2.627	1.791	2.87	3.048	2.987	2.078	3.323

### Volatile oil components and disease-related target mapping

**Figure 5.**
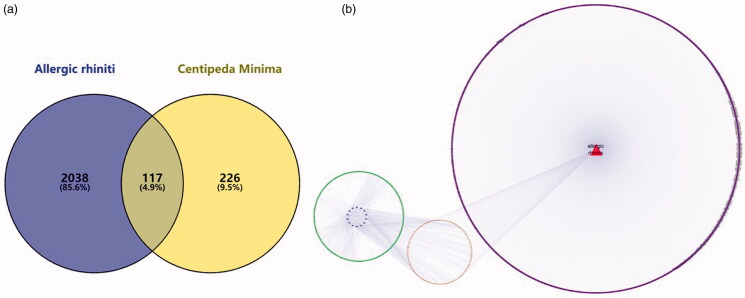
(a) Composition target and disease target Venn diagram. (b) Ingredient-target-disease (The blue on the left represents the component, the green is the target of the component, the red on the right is the disease name, the purple is the disease target, and the orange in the middle represents the component target and the disease common target).

The obtained component targets and disease-related targets were introduced into Veeny software. Consequently, 117 intersecting genes were obtained as shown in Figure 5(a). The component-target-disease network diagram is shown in Figure 5(b) Blue color on the left represents the 15 components. Green color represents the corresponding target of the component. Orange color in the middle indicates the intersection target of the component and the disease, while purple on the right represents the target of the disease only.

### Protein interaction network

**Figure 6.**
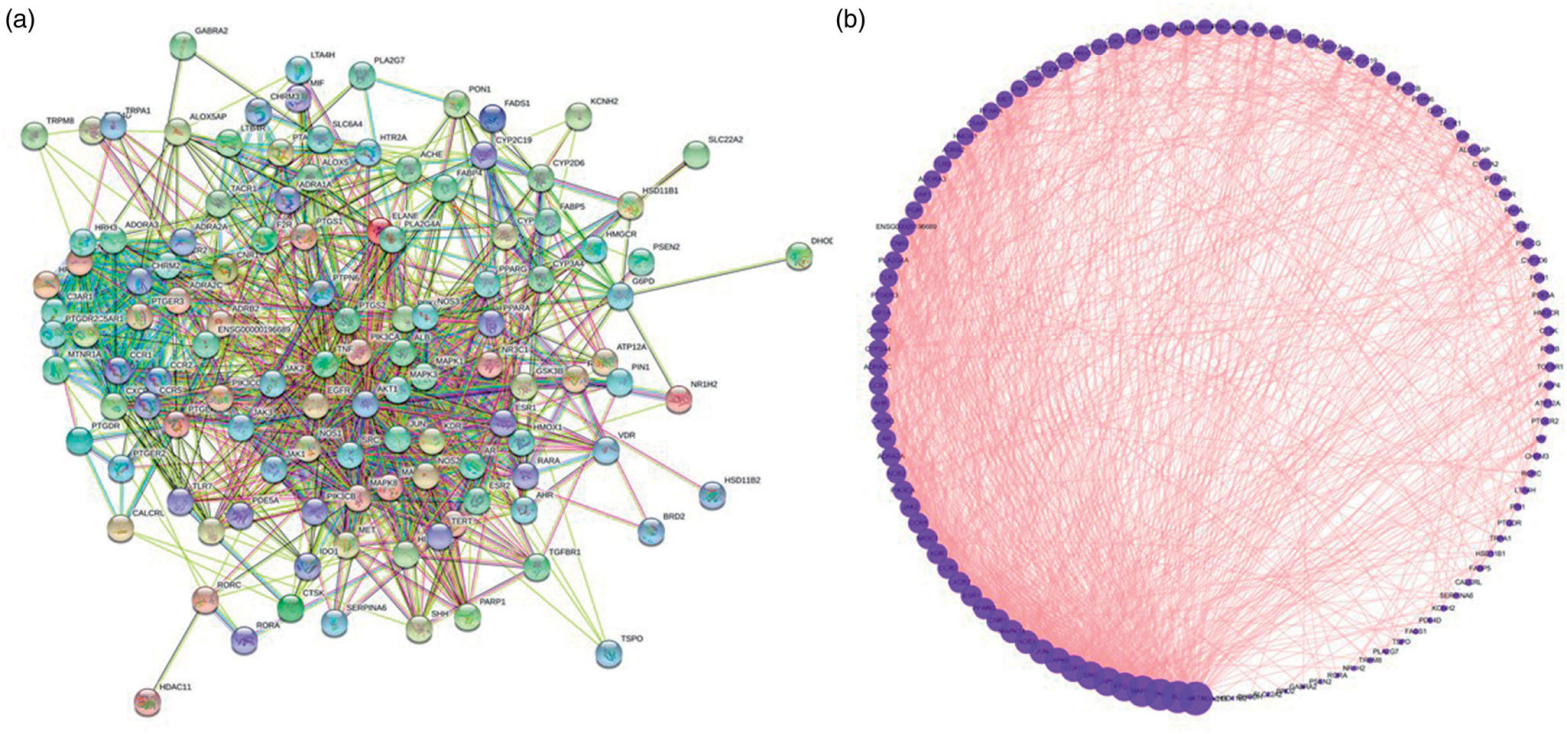
Protein interaction.

To analyze protein interactions, 117 intersection targets were imported into the STRING platform as shown in Figure 6(a). Red color indicates evidence of fusion, green - evidence of proximity, yellow - evidence collected via text mining, light blue - evidence collected via database search, blue - evidence of coexistence, black - evidence of co-expression, and purple - experimental evidence. The analysis results were imported into STRING and Cytoscape 3.7.1. The resulting protein interaction diagram is shown in Figure 6(b). In this figure, the size of the circle changes according to the degree value of each protein, higher degree value indicates that a protein participates in more pathway interactions. According to the degree and betweenness centrality values, three proteins were selected, including tumor necrosis factor (TNF), prostaglandin-endoperoxide synthase 2 (PTGS2), and mitogen-activated protein kinase 3 (MAPK3).

### GO analysis and KEGG analysis

**Figure 7.**
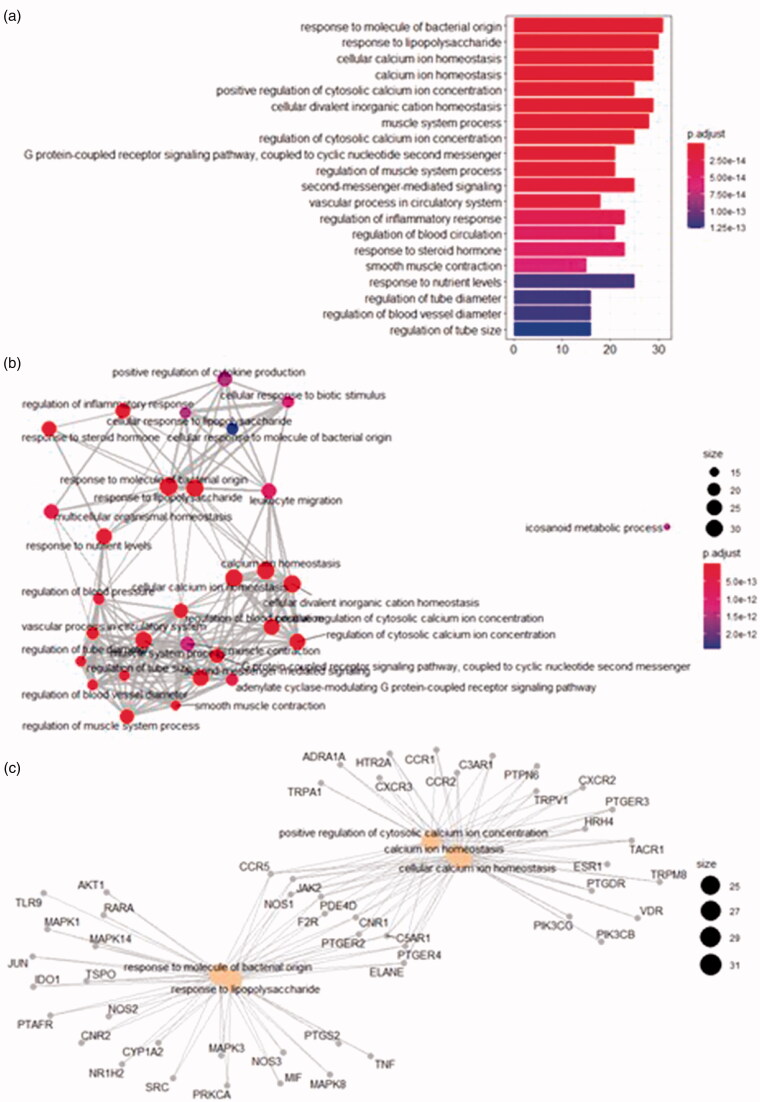
BP analysis. (a) top 20 pathways. (b) pathway relationship. (c) core pathway.

ClusterProfiler package in R language was used to perform GO and KEGG analysis on the intersection targets. The GO analysis results show that the biological process (BP) was associated with 1753 pathways, including response to molecule of bacterial origin, regulation of inflammatory response, and cellular calcium ion homeostasis, these entries reflect that genes are involved in related biological processes in vivo and cooperate in the treatment of allergic rhinitis. The enrichment analysis of the top 20 pathways and corresponding *p*-value are shown in Figure 7(a). The horizontal axis indicates the number of genes in the BP term. The vertical axis describes the used terms. The color changes from blue to red according to p values adjusted from smaller to larger. Figure 7(b), each node represents an enriched BP entry and the node size corresponds to the rich number of genes under the entry. Indicating various relationships between entries (core pathway) as shown in Figure 7(c). The grey dots represent genes and the yellow - entry names. The figure demonstrates enriched targets in each pathway and shows that *C. minima* is involved in multiple biological processes associated with allergic rhinitis.

**Figure 8.**
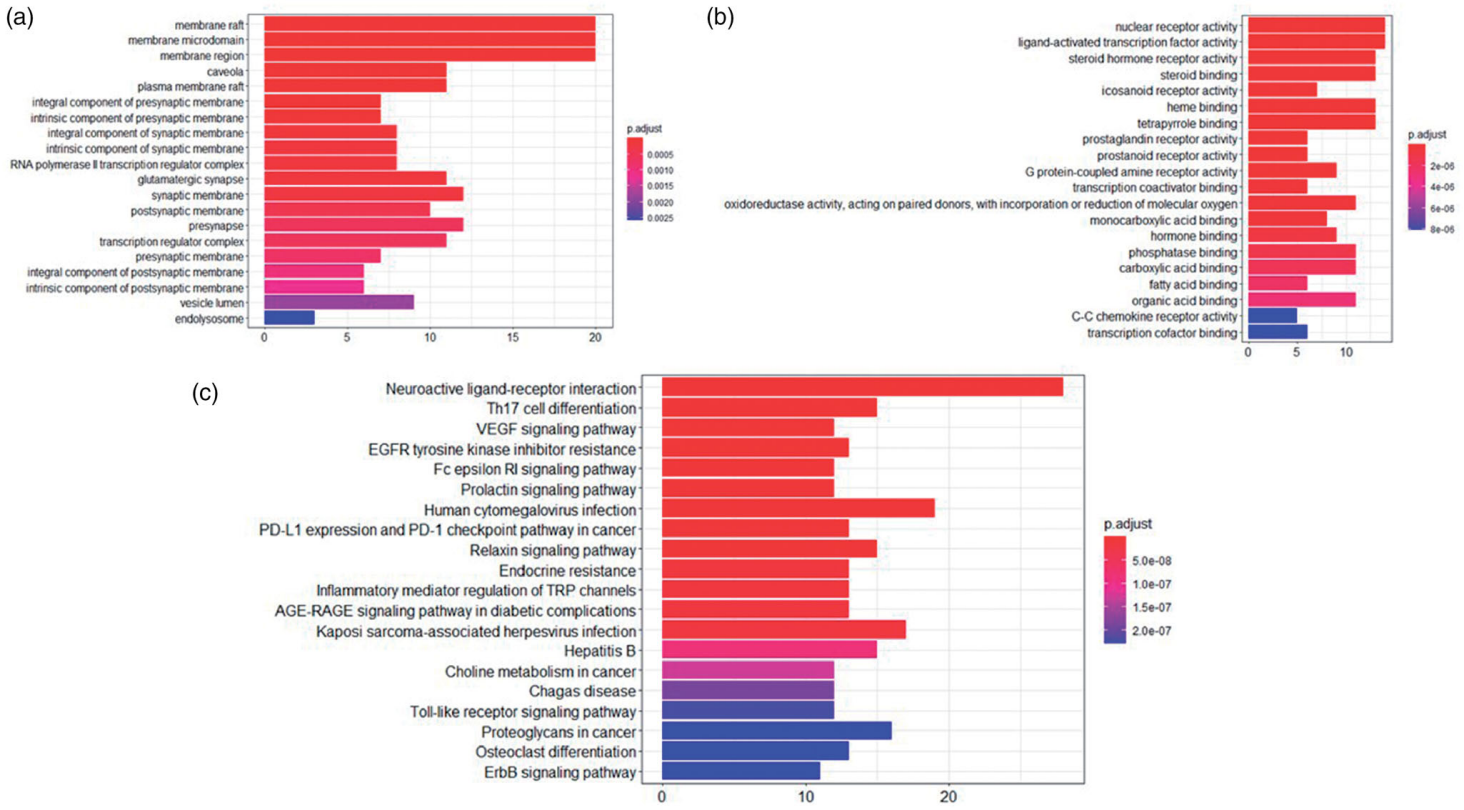
(a) CC analysis. (b) MF analysis. (c) KEGG pathway.

Our data analysis detected 37 cellular component (CC) pathways liked to membrane region, an integral component of the presynaptic membrane, transcription regulator complex, and others pathways involved in the pathology of allergic rhinitis. Figure 8(a) shows the enrichment analysis results of each pathway according to its significance (*p* value).

Molecular function (MF) analysis defined 146 pathways including nuclear receptor activity, ligand-activated transcription factor activity, and G protein-coupled amine receptor activity. It has been shown that regulated sequence-specific DNA binding is involved in the occurrence of allergic rhinitis. Figure 8(b) shows the enrichment analysis results for the top 20 pathways according to their *p*-values.

KEGG analysis identified 137 related pathways. Among them, 28 target proteins were shown to participate in neuroactive ligand-receptor interaction, 15 target proteins are involved in Th17 cell differentiation, and 12 target proteins regulate VEGF signaling pathway. The data analysis shows that *C. minima* active ingredients are associated with the activation of multiple pathways that can be potentially linked to allergic rhinitis. Figure 8(c) shows the top 20 pathways identified according to their *p* values. According to the enrichment analysis results and the existing literature reports, the second Th17 cell differentiation pathway is involved in the development of allergic rhinitis and is an important pathway in allergic rhinitis (Li et al. 2020; Qin et al. 2020).

### Results of molecular docking analysis

**Figure 9.**
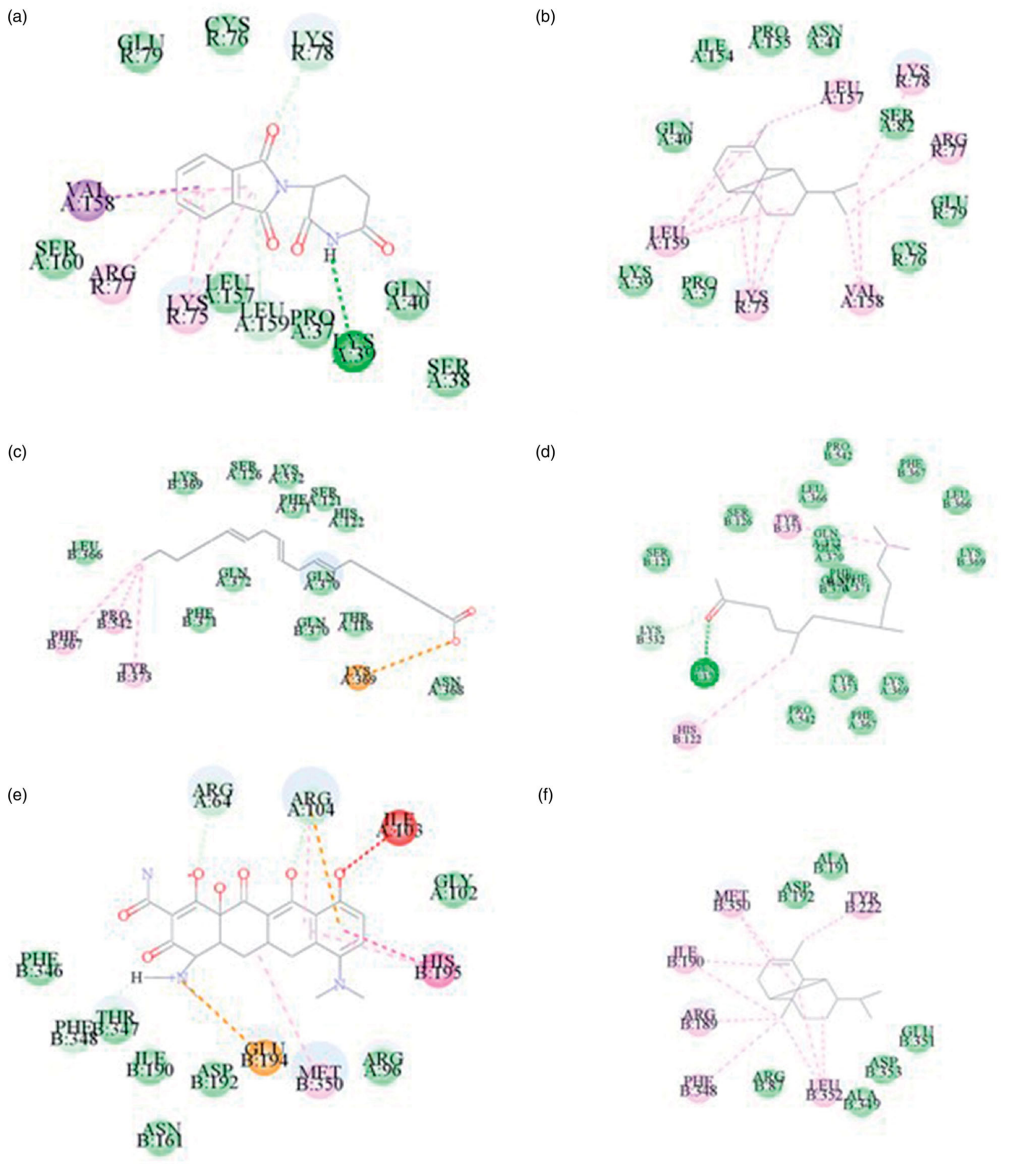
Molecular docking. (a) 5426 (Thalidomide) with TNF 2 D diagram. (b) 19725 (Copaene) with TNF 2 D diagram. (c) 5280581 (Dihomo-γ-linolenic acid) with PTGS2 2 D diagram. (d) 10408 (2-Pentadecanone, 6,10,14-trimethyl-) with PTGS2 2 D diagram. (e) 54675783 (Minocycline) with MAPK3 2 D diagram. (f) 19725 (Copaene) with MAPK3 2 D diagram.

TNF, PTGS2, and MAPK3 were identified as the top three proteins according to the calculated degrees and betweenness centrality values. These proteins were docked to their corresponding *C. minima* components and positive drugs. The docking analysis showed that these three proteins can be stably docked with the *C. minima* components. Notably, MAPK3 protein docking score was higher than that of the positive control drug. TNF protein docking score was lower than that of the positive control drug. The docking scores are shown in Table 3, and the docking analysis results are shown in Figure 9. The figure shows the relationship between key amino acid residues and molecular functional groups. Amino acid residues are represented by small discs. Amino acid names are abbreviated on the discs. Dark green represents hydrogen bonds; light green – carbon-hydrogen bonds; and purple – alkyl groups. Dark purple indicates π-π stacking; orange – electron-withdrawing; and the dotted line – the interaction between the acceptor residue and the ligand atom.

**Table 3.** Molecular docking scores.

Protein	Ligand	PubChem CID	LibDock Score
TNF	Thalidomide（Positive drug）	5426	86.6862
	Copaene	19725	80.7849
	Petasitene	636697	71.3039
	10*S*,11*S*-Himachala-3(12),4-diene	14038471	64.6302
PTGS2	Dihomo-gamma-linolenic acid （Positive drug）	5280581	127.277
	2-Pentadecanone, 6,10,14-trimethyl-	10408	111.073
MAPK3	Minocycline（Positive drug）	54675783	70.8758
	Copaene	19725	82.6413
	trans-.alpha.-Bergamotene	6429302	80.8744
	Petasitene	636697	63.4729
	Modephene	11030947	61.844

### H&E staining results

**Figure 10.**
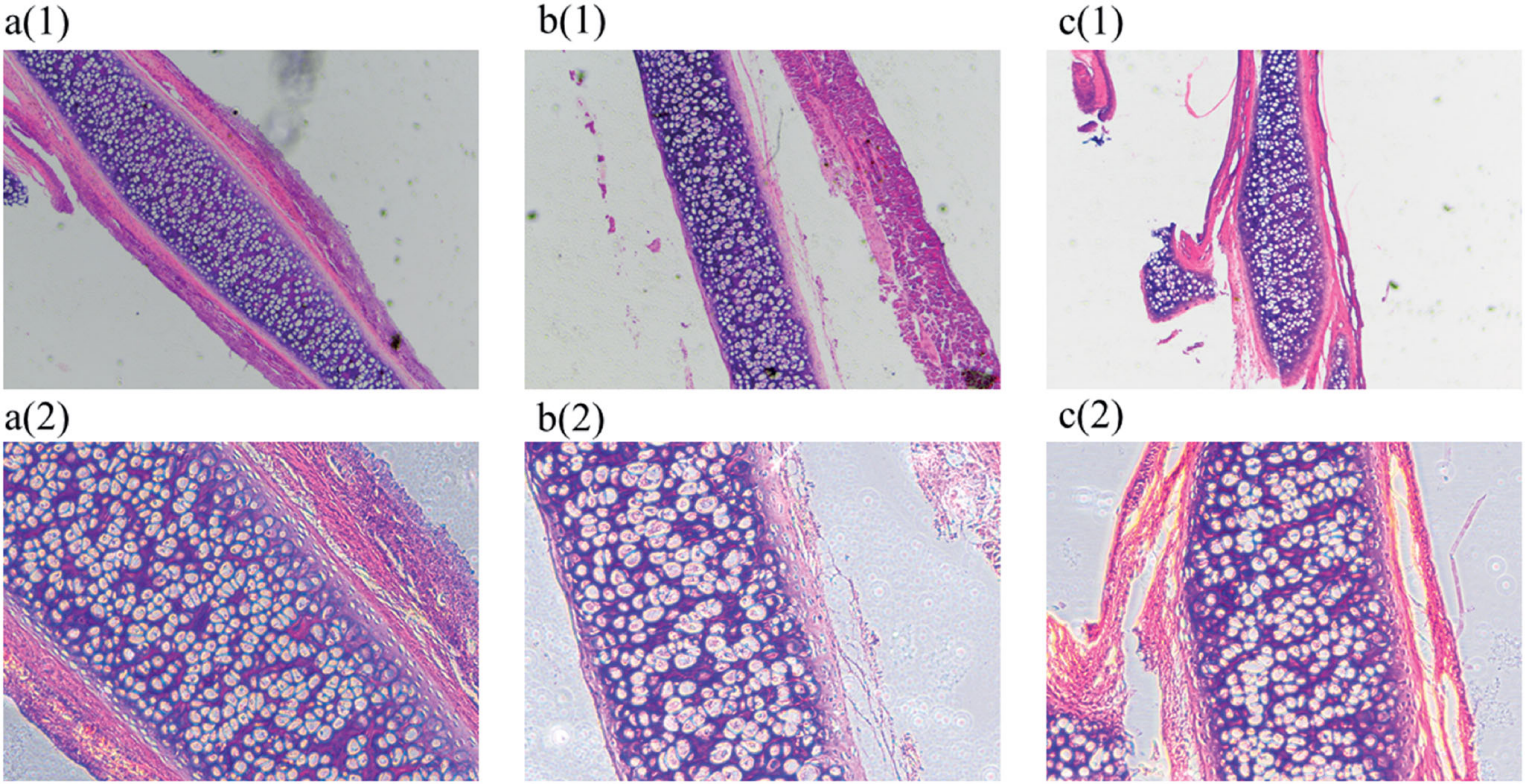
Haematoxylin-eosin stain (a) (1)(2) is the blank group 4 × 10 and 10 × 10, (b) (1)(2) is the model group 4 × 10 and 10 × 10, (c) (1)(2) is the therapy group 4 × 10 and 10 × 10).

Histopathological rat tissues analysis demonstrated that nasal mucosa epithelium in the blank control group was not damaged. No inflammatory cell infiltration was observed in the submucosa of blank control rats. Alternatively, in the disease model group, a large amount of nasal mucous cilia fell off and nasal epithelium was damaged. Tissues samples indicated gland hyperplasia and swelling, interstitial edema, and interstitial inflammatory cell infiltration in the affected animals. In the *C. minima* extract-treated rats, the nasal mucosal damage was significantly lower, with lower glandular hyperplasia, and lower level of inflammatory cells infiltration in the interstitial cell layers. The results are shown in Figure 10.

### ELISA results

**Figure 11.**
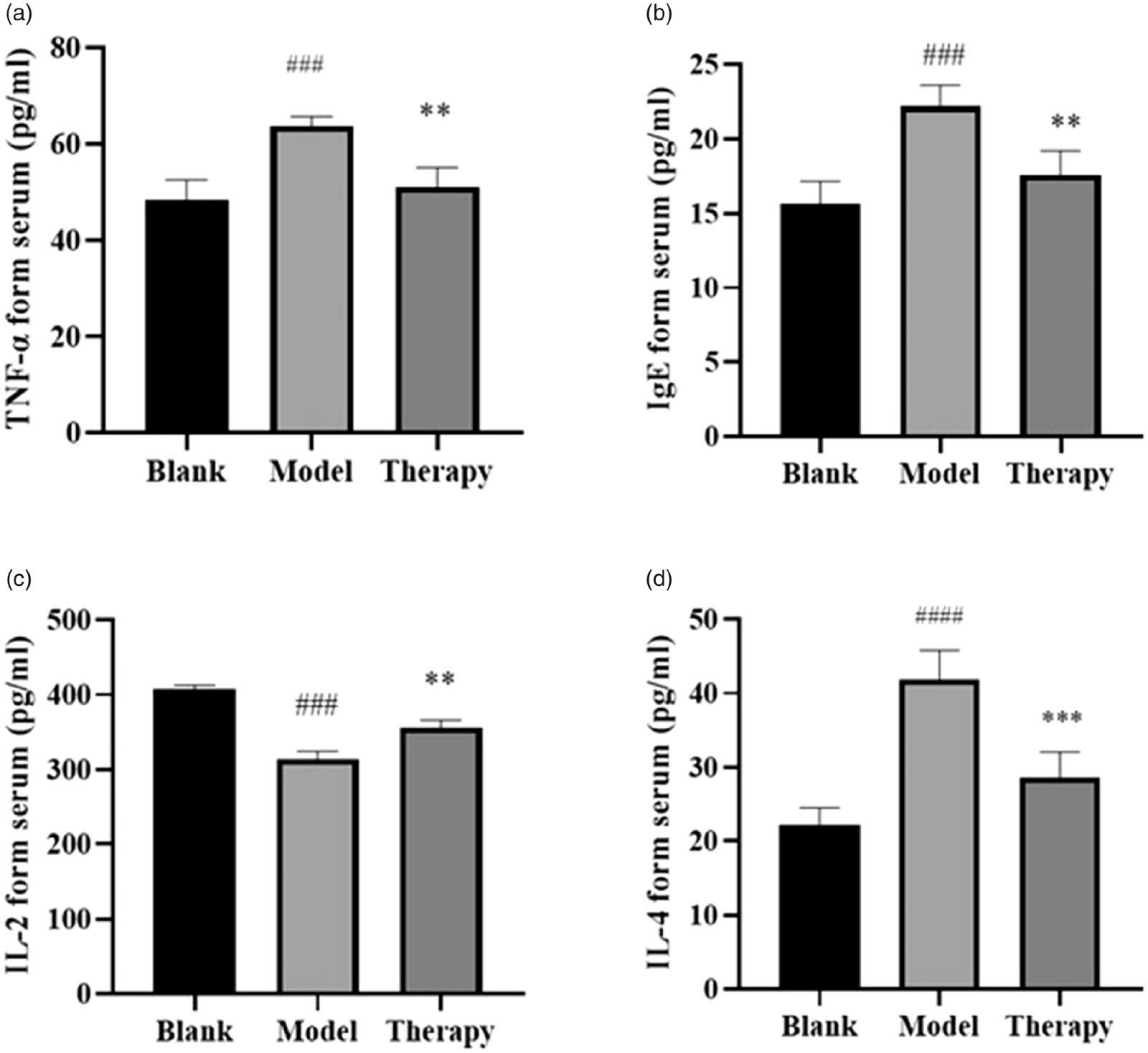
ELISA results. (a) TNF-α. (b) IgE. (c) IL-2. (d) IL-4 ####*p* < 0.0001, ###*p* < 0.001 versus blank group; **p < 0.01, ***p < 0.001 versus model group.

Interleukins, as second messengers, can activate and regulate immune cells and participate in activation of many inflammatory pathways including Th17 cell differentiation. For instance, TNF promotes cell proliferation and differentiation. As an important inflammatory factor, IgE is the reference marker for the occurrence and development of allergic rhinitis. We detected the levels of inflammatory factors (IL-4, TNF- α, IgE) and anti-inflammatory factor IL-2 in serum to further verify the pathway and targets of the network pharmacology. ELISA was used to determine the contents of TNF, IL-2, IgE, and IL-4 in rat serum (Figure 11). Compared with the blank group, the TNF content of the model group was significantly higher (*p* < 0.001), while the TNF level in the treatment group was significantly lower than that in the model group (*p* < 0.01). Compared with the blank group, the IL-2 content in the model group was significantly lower (*p* < 0.001). IL-2 level in the treatment group was significantly higher (*p* < 0.01) compared with the model group. IL-4 content in the model group was significantly increased (*p* < 0.001) compared with the blank group. IL-4 level in the treatment group was significantly decreased (*p* < 0.001) compared with the model group. The IgE analysis showed that its content in the model group was significantly higher than that of the blank group (*p* < 0.001). IgE levels in the treatment group were significantly lower than those in the model group (*p* < 0.01). Therefore, *C. minima* can inhibit inflammatory factors and promote anti-inflammatory factors. This effect indicates the suitability of the plant for the treatment of allergic rhinitis.

### Immunohistochemistry

**Figure 12.**
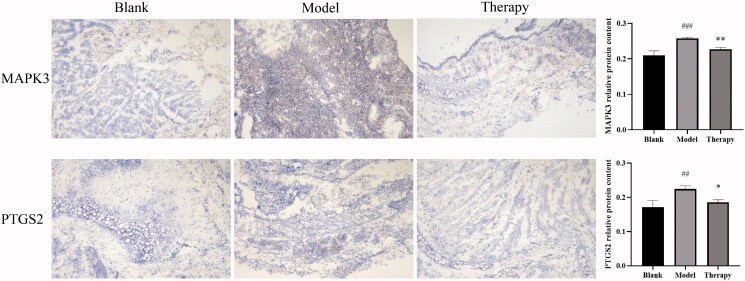
Immunohistochemistry ###*p* < 0.001, ##*p* < 0.01 versus blank group; *p < 0.05, **p < 0.01 versus model group.

The results of immunohistochemistry are shown in Figure 12, the expression rate of PTGS2 and MAPK3 in inflamed tissues was significantly higher than that in normal tissues. Compared with the blank group, the average optical density of PTGS2 and MAPK3 proteins in the model group was significantly increased (*p* < 0.01). Compared with the model group, the average optical density value of PTGS2 and MAPK3 protein in the volatile oil-administered group was significantly lower (*p* < 0.05).

## Discussion

Allergic rhinitis is a common type of allergic inflammatory disease triggered by environmental allergens and mediated by IgE (Kim et al. 2004). The pathology incidence is marked by an upward trend globally. Allergic rhinitis affects daily activities and represents a significant health problem (Huang H et al. 2019). *C. minima* is a known medicinal herb used to treat allergic rhinitis (Yu et al. 2001). However, the mechanisms of *C. minima*’s effects remain unclear.

In this study, we found the differences in the composition and content of volatile oil in *C. minima* collected in different areas. The difference may be associated with the variety in area elevations above sea level, climate temperature, soil types, and cultivation methods, all of which have certain effects on the growth and internal quality of medicinal materials (Neffati et al. 2009). However, no significant differences in composition and content were found between Jiangxi and Hubei, Shanxi, and Sichuan. Suggestively, the similarity is observed because those places are geographically adjacent, and shown no significant differences in climate and species. The significant differences between Hubei and Guangxi, Henan, and Guangdong may be explained by the geographical distance between the places, and the greater differences in climate temperature and altitude, which may be associated with the difference in the quality of volatile oil. In this experimental study, pharmacological network analysis was used to determine the protein interactions and activation of pathways associated with allergic rhinitis and triggered by the volatile components of *C. minima* from seven different geographic areas. The main pathways associated with allergic rhinitis and *C. minima* were linked to neuroactive ligand-receptor interaction. The results of KEGG analysis show that neuroactive ligand-receptor interaction is defined by collective signaling of all receptors and ligands related to specific extracellular and extracellular signaling pathways on the plasma membrane, including Th17, VEGF, and TNF signaling. Th17 cell differentiation pathway is a unique pro-inflammatory CD4 + T cell pathway, which plays an important role in the pathophysiological process of allergic rhinitis (Liu et al. 2018). VEGF is a marker of endothelial injury and can be induced by inflammatory factors such as TNF and other interleukins (Persad et al. 2012; Zhang et al. 2021). Our analysis identified 117 proteins that may be involved in the generation of synergistic anti-inflammatory effects and impact the described above 137 inflammation-related pathways.

Screening for the key target proteins that act on the disease indicated that these proteins are TNF, PTGS2, and MAPK3. It is known from the literature that TNF is a multi-effect proinflammatory cytokine, which can participate in the activation of chemotaxis in neutrophils and eosinophils to vascular endothelial cells and induce the production of pro-inflammatory factors in vascular endothelial cells and fibroblasts in allergies. TNF plays an important role in the occurrence and development of disease response and treatment outcome (Zhang et al. 2014; Zhou et al. 2019). PTGS2 is a prostaglandin, an inflammatory mediator. PTGS2 regulates eosinophil leukocyte migration during inflammatory responses (Zhe et al. 2020). MAPK3 is one of the MAPK family effectors. MAPK is an important signal transducer from the cell surface to the nucleus. By phosphorylating downstream products such as kinases or transcription factors, MAPK regulates the expression of related genes, the transcription, and translation of inflammatory factors, and plays an important role in the activation, maturation, differentiation, and immune response of inflammatory cells (Huang et al. 2010; Liu et al. 2015). Molecular docking analysis was performed on the above key proteins. The docking analysis results show that the target protein can bind the *C. minima* components, and the docking score is better or slightly lower than that of the positive drug control. At the same time, immunohistochemistry was used to semi-quantitatively analyze the PTGS2 and MAPK3 proteins in pathological tissues, and further corresponded to the software results. The results showed that the positive expression rate of PTGS2 and MAPK3 in the inflammatory tissues of the model group was significantly higher than that of the blank group and the pathological tissues of the administration group. During the progression of allergic rhinitis, IgE was described as the central trigger that switches the release of inflammatory substances causing nasal diseases. Accordingly, IgE is an important reference marker for the emergence and development of the disease (Sadeghi et al. 2020). IL-4 is also a pro-inflammatory cytokine (Wu et al. 2021), Alternatively, IL-2 can inhibit IgE production, and play an anti-inflammatory role (Niu et al. 2020). The change in serum cytokine content is an important indicator that reflects the evolution of allergic rhinitis. Our data shows that after the initiation of allergic rhinitis, the contents of IgE and IL-4 were decreased and the content of IL-2 was increased after administration of *C. minima* components in the model animals. Our findings suggest that C. minima components played a therapeutic effect via regulation of TNF, IL-2, IL-4, and IgE levels.

Through animal experiments, we also found that *C. minima* can significantly improve the proliferation of eosinophils and other inflammatory cells, as well as reducing the pathological changes of nasal mucosa. *C. minima* oils appear to coordinate the reduction of rhinitis through multiple targets and multiple pathways, which can provide a basis for the further development and medicinal utilization of these components. However, the continuous improvement of network technology and the real-time update of the database indicate the necessity for further testing. Therefore, the targets of *C. minima* treatment for allergic rhinitis should be verified in future research.

## Conclusion

In this study, the optimum conditions for the extraction of volatile oil from *C. minima* by steam distillation were obtained, the contents of volatile oil were different in 7 geographic areas. The experimental results of rhinitis model rats are consistent with the results of network pharmacological data analysis. Molecular docking shows that TNF, PTGS2, and MAPK3 have a good affinity for the components. Immunohistochemistry further shows that the protein content of PTGS2 and MAPK3 in the administration group is significantly reduced, and it can reduce the serum inflammatory factor TNF-α, IL-4, and IgE, increase the concentration of anti-inflammatory factor IL-2, by regulating neuroactive ligand-receptor interaction, Th17 cell differentiation, VEGF signaling pathway and other biological pathways play a role, the volatile oil of *C. minima* has a significant effect on the treatment of allergic rhinitis and is multi-target and multi-channel.
